# F-18 FDG PET brain imaging in symptomatic arthroprosthetic cobaltism

**DOI:** 10.1007/s00259-019-04648-2

**Published:** 2019-12-20

**Authors:** Robert L. Bridges, Christina S. Cho, Marc R. Beck, Bradford D. Gessner, Stephen S. Tower

**Affiliations:** 1 Aegis Imaging Consultants, LLC, P.O. Box 751, 170 Cervin Circle, Girdwood, AK 99587 USA; 2 Tower Joint Replacement Clinic, Inc., Anchorage, AK USA; 3 Turnagain Radiology Associates, LLC, Anchorage, AK USA; 4 EpiVac Consulting Services, Anchorage, AK USA

**Keywords:** Arthroprosthetic, Cobaltism, Encephalopathy, Toxicity

## Abstract

**Purpose:**

Imaging studies of cobalt toxicity from cobalt-chromium alloy arthroprosthetics have focused on the local intra-articular and peri-articular presentation from failing joint replacements. Most studies investigating neurological findings have been small case series focused on the clinical findings of memory loss, diminished executive function, tremor, hearing and vision loss, depression, and emotional lability. This study utilizes software-based quantitative analysis of brain metabolism to assess the degree of hypometabolism and areas of susceptibility, determine if a pattern of involvement exists, and measure reversibility of findings after prosthetic revision to cobalt-free appliances.

**Methods:**

Over 48 months, 247 consecutive patients presenting to an orthopedic clinic with an arthroprosthetic joint containing any cobalt-chromium part were screened with whole blood and urine cobalt levels. A clinically validated inventory of 10 symptoms was obtained. Symptomatic patients with a blood cobalt level above 0.4 mcg/L or urine cobalt greater than 1 mcg/L underwent F-18 FDG PET brain imaging. Analysis was performed with FDA-approved quantitative brain analysis software with the pons as the reference region. Control group was the normal brain atlas within the software.

**Results:**

Of the 247 consecutively screened patients, 123 had blood and urine cobalt levels above the threshold. The 69 scanned patients had statistically significant regional hypometabolism and higher symptoms inventory. Fifty-seven patients were retained in the study. Distribution of hypometabolism was in descending order: temporal, frontal, Broca’s areas, anterior cingulate, parietal, posterior cingulate, visual, sensorimotor, thalamic, and lastly caudate. Metal-on-metal (MoM) and metal-on-plastic (MoP) joint replacements produced similar patterns of hypometabolism. Of 15 patients with necessary revision surgery, 8 demonstrated improved metabolism when later re-scanned.

**Conclusion:**

All scanned patients had regions of significant hypometabolism. Neurological toxicity from elevated systemic cobalt levels following arthroprosthetic joint replacement has a pattern of regional susceptibility similar to heavy metals and solvents, differing from classical dementias and may occur at blood and urine cobalt levels as low as 0.4 mcg/L and 1 mcg/L, respectively. Presently accepted thresholds for cobalt exposure and monitoring may need revision. Quantitative F-18 FDG PET brain imaging may aid in the decision process for treatment options and timing of possible medical versus surgical intervention.

## Introduction

Modern hip, shoulder, and knee joint replacements employ metal, ceramic, or plastic materials to replace one or both articular surfaces. Iron (FeCrNi), cobalt (CoCr), and titanium (TiAlV) are the base metals of arthroprosthetic alloys. Of these, only cobalt is notably toxic. [1–5] Globally, about 40 million patients have replaced hip, shoulder, or knee with about half residing in the USA. [6, 7] Most have at least one CoCr component. [8]

**Fig. 1:**
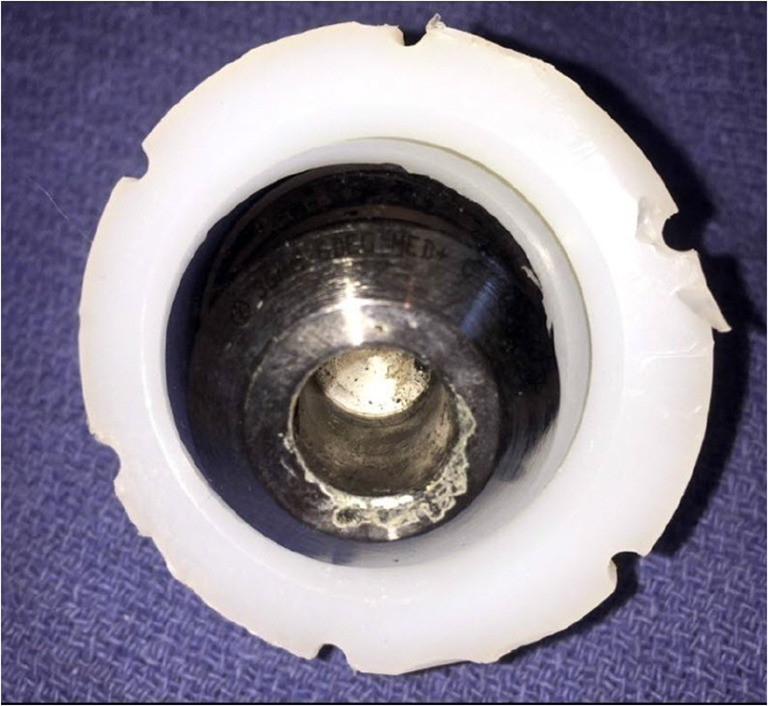
Corrosion of chromium-cobalt head in metal-on-plastic (MoP) hip prosthesis

Periprosthetic CoCr metallosis produced by wear or corrosion of CoCr articular components (heads or sockets) or at the taper junctions (Fig. 1) of articular and non-articular components (stems, necks, or trays) may result in painful or painless periprosthetic bone and tissue inflammation, necrosis, and pseudotumor formation. [6–12] Collectively, these radiographic and clinical symptoms are called Adverse Reaction to Metal Debris (ARMD). [10] Patients may also react with an immune response to CoCr implants by direct attack of leukocytes resulting in ARMD and systemically from circulating cobalt in the absence of significant implant wear or taper corrosion. [13]

Systemic cobalt toxicity (cobaltism) most commonly affects the nervous, endocrine, and cardiovascular systems, which may result in deafness, blindness, peripheral neuropathy, thyropathy, polycythemia, cardiac failure, and, in extreme cases, death. [1–4] Profound systemic toxicity results when a fractured ceramic hip implant is revised to a CoCr femoral head, or when a CoCr ball and socket articulate (Metal-on-Metal Hip). [4] Nominal cobalt exposure from wear or corrosion of modular CoCr implants or CoCr articular surfaces can cause a subtler presentation of neurologic, psychiatric, and constitutional maladies, easily confused with aging, and similar to the vocational manganese toxicity. [4, 12, 14, 15]

F-18 FDG PET brain imaging has become instrumental in early detection and monitoring of neurodegenerative disease. [16–21] In 1991, Callender utilized visual interpretation of SPECT and PET imaging to identify regions of brain injury from exposure to organic solvents. [22] Since then, FDA-approved analytic software has been developed that can provide quantitative analysis of regional brain metabolism. Such software provides a reproducible and objective dataset without inter-observer variability in processing and interpretation of the scan. [23]

## Method

The study underwent IRB review and approval for inclusion of data from redacted patient files. Over a period of 48 months, 247 patients presenting consecutively to an orthopedic clinic with a presumed cobalt-chromium alloy prosthesis were systematically screened with a symptom inventory along with whole blood and urine cobalt levels. Cobalt is an intra-cellular toxin compromising mitochondrial function. The assay for cobalt was referenced to whole blood as a cellular analog versus serum levels which vary with albumin levels. Specimens for cobalt analysis were collected in metal-free EDTA trace elements tube. Analysis of samples was conducted by inductively coupled plasma/mass spectrometry. Thresholds from laboratory studies were 0.4 mcg/L for blood cobalt or 1 mcg/L for urine cobalt. These laboratory values are greater than the 95th percentile for the populace at large.

Cobalt Symptom Score was assigned by one author at the time of the clinic visit during which the patient was enrolled into the study, nearly always before blood or urine cobalt level was known. All patients consented to be studied. Early in the study, 10 of the patients who underwent cobalt symptom inventory score also underwent neurocognitive testing by a clinical psychologist and 16 were administered the Montreal Assessment of Cognitive Function (MoCA) by another author. These evaluations were discontinued as they were not perceived to be helpful in the clinical decision-making processes and resources for continuance were limited.

For the symptom inventory, patients were questioned about 10 symptoms: forgetfulness, fatigue, moodiness, imbalance, tremor, poor sleep, numbness or weakness, deafness or tinnitus, global pain, and non-refractive blindness. Maximum score was 14 with one point per symptom except a score of 0–3 for tremor and 0–3 for peripheral neuropathy. A symptom was only scored if it occurred after the at-risk joint implantation and if it was beyond the patient’s or family member’s expectation for normal aging. Information was also gathered on the patient’s potential exposure to heavy metals and organic solvents.

Of the 247 patients, 123 had blood and/or urine cobalt levels above threshold. Of the 123 patients, 69 underwent F-18 FDG PET brain scans as part of their neurological examination. The barrier of third-party reimbursement randomly constrained the number of patients scanned. Many third-party payers and governmental agencies had limited indications for PET brain imaging. Some confined scanning to differentiating Alzheimer’s from Pick’s Disease. Other carriers required peer-to-peer review for approval. Finally, some patients chose to underwrite the examination personally. The best single indication for approval was “exposure to toxins.”

Studies were performed at three imaging centers. The scanners were GE Discovery ST (General Electric, Milwaukee, WI), Philips Gemini 16 (Philips Medical Systems, Bothell, WA), and Siemens Biograph MCT (Siemens Medical Malvern, PA). Fasting patients were injected intravenously with about 370 megabecquerels of F-18 FDG, followed by 1 h uptake in a dim quiet room and then were scanned using standard default protocol per each scanner.

Two board-certified diagnostic radiologists reviewed the CT portion of the PET/CT scans to identify any exclusionary neuro-anatomic abnormalities such as stroke, asymmetric loss of volume, or significant regional atrophy. A board-certified Nuclear Medicine Physician conducted the processing of the PET/CT images with NeuroQ (Syntermed Atlanta, GA) using a Mirada XD3 workstation (Mirada Medical Oxford, England) for connectivity. NeuroQ is an FDA-approved program to analyze metabolism of the brain and statistically compare the patient’s scan with a whole brain atlas matched for age and sex. NeuroQ separates the brain into 240 regions for comparison and then combines related regions into 47 statistically significant cluster areas. Threshold for abnormality is ± 1.65 standard deviations.

Default settings within the software were used. Upon completion, the analysis was set to the reference region of the pons. The pons was chosen for the reference region as potentially having the best resiliency to toxicity. Since the whole brain is exposed to cobalt, “whole brain” as a reference region was thought to potentially create spuriously underestimated hypometabolism. Likewise, the cerebellum has been reported as hypometabolic by SPECT in mercury, bromine, and manganese poisoning [24]. Data output was recorded for hypometabolism and included number of regions affected, summed score of those regions, along with number of abnormal clusters, their location, and standard deviation. Since the software analysis was performed with default settings and statistical output was independent of visual interpretation, the findings were considered free of observer variability.

The control group was the atlas of normal brain metabolism within the NeuroQ program. The patients’ primary differences with the general population were joint surgery and general anesthesia. Neither orthopedic surgery nor general anesthesia is presently considered detrimental to long-term brain metabolism. Patients with blood or urine levels below the 95th percentile were not considered as a required control group separate from the normal brain atlas. Clark used advanced MRI sequences and software analysis to compare asymptomatic CoCr alloy hip-implanted patients with minimally elevated serum cobalt levels against a reference group of total hip replacement patients with normal cobalt levels. Subtle structural changes in the brain were only found in the patients with elevated cobalt. [25]

Patients were arranged by increasing degree of hypometabolism according to summed hypometabolic scores, number of regions affected, and number of hypometabolic clusters. From this, an arbitrary separation into four tiers was made to assess progression of regional involvement (Table 1).

**Table 1:** Range of hypometabolic scores, regions, and clusters

Tiers by summed hypometabolism	Number of patients	Range of summed score of hypometabolic regions	Number of hypometabolic regions	Number of hypometabolic clusters
1 (0 to − 50)	14	− 17.7 to − 46	7 to 23	1 to 6
2 (− 50 to − 120)	13	− 54 to − 110	25 to 45	3 to 10
3 (− 120 to − 220)	17	− 119 to − 218.3	49 to 84	11 to 22
4 (>− 220)	13	− 221.7 to − 381.7	82 to 134	17 to 28

There are no preceding published articles utilizing F-18 FDG PET brain imaging to assess brain metabolism in patients with systemically elevated levels of cobalt from artificial joints and presenting with clinical signs of neurotoxicity. As such, the evaluation of data rested, in part, on published articles dealing with other forms of neurotoxicity from organic chemicals and metals such as mercury and manganese. [24, 26–28]

## Results

Of the 57 retained patients, 17 had metal-on-metal (MoM) hips, 38 had metal-on-plastic (MoP) hips, 1 had a ceramic-on-plastic (CoP) hip, and one patient had an all cobalt alloy metal knee prosthesis with the demographics for the four tiers displayed in Table 2. Symptom inventory for the remaining 57 patients is listed in Table 3. In all, 12 patients were excluded from the main study. Three patients were excluded due to abnormal neuro-anatomic findings on CT although one was retained who required surgery and would be suitable for pre- and post-revision assessment. Six patients whose post-revision scans had increased hypometabolic findings were excluded. Particularly for this subgroup, there was no way to differentiate ongoing cobalt toxicity, potential end-stage progression of encephalopathy due to cobalt, and/or a confounding progressive neurodegenerative process from solely cobalt toxicity. An additional patient was removed as a global highly hypometabolic outlier. Two patients were diagnosed with Alzheimer’s Disease.

**Table 2:** Demographics per tier for age, phenotype, duration of implant, blood and urine cobalt (mcg/L), and type of cobalt alloy prostheses

Tier	Age	M/F	Duration (year)	Co (blood)	Co (urine)	MoM	MoP	CoP	Knee
1	51–89	8/6	2.8 to 27.5	0.4 to 6.3	0.7 to 29.8	6	8	0	0
2	54–79	2/9	5 to 20	0.4 to 20.2	1.4 to 55.4	2	11	0	0
3	62–83	12/5	2.8 to 19.7	0.3 to 9.4	0.5 to 31.8	4	11	1	1
4	59–84	9/4	2.1 to 20.1	0.4 to 5.7	0.9 to 49.1	5	8	0	0

**Table 3:** Symptoms inventory for patients with normal cobalt levels versus those scanned

Symptom	Normal cobalt level (*n* = 110)	Symptomatic in study (*n* = 57)
Fatigue	16%	72%
Sleep disorder	5%	51%
Mood	13%	60%
Forgetfulness	18%	75%
Balance	17%	54%
Tremor	28%	82%
Deafness/tinnitus	16%	44%
Numbness/weakness	15%	51%
Global pain	7%	39%
Non-refractive blindness	2%	23%

**Fig. 2:**
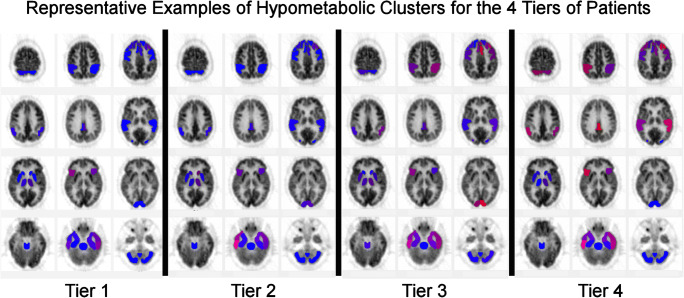
Representative NeuroQ scans for each tier of patients. Blue is normal with shades toward red indicating increasing hypometabolism

**Fig. 3:**
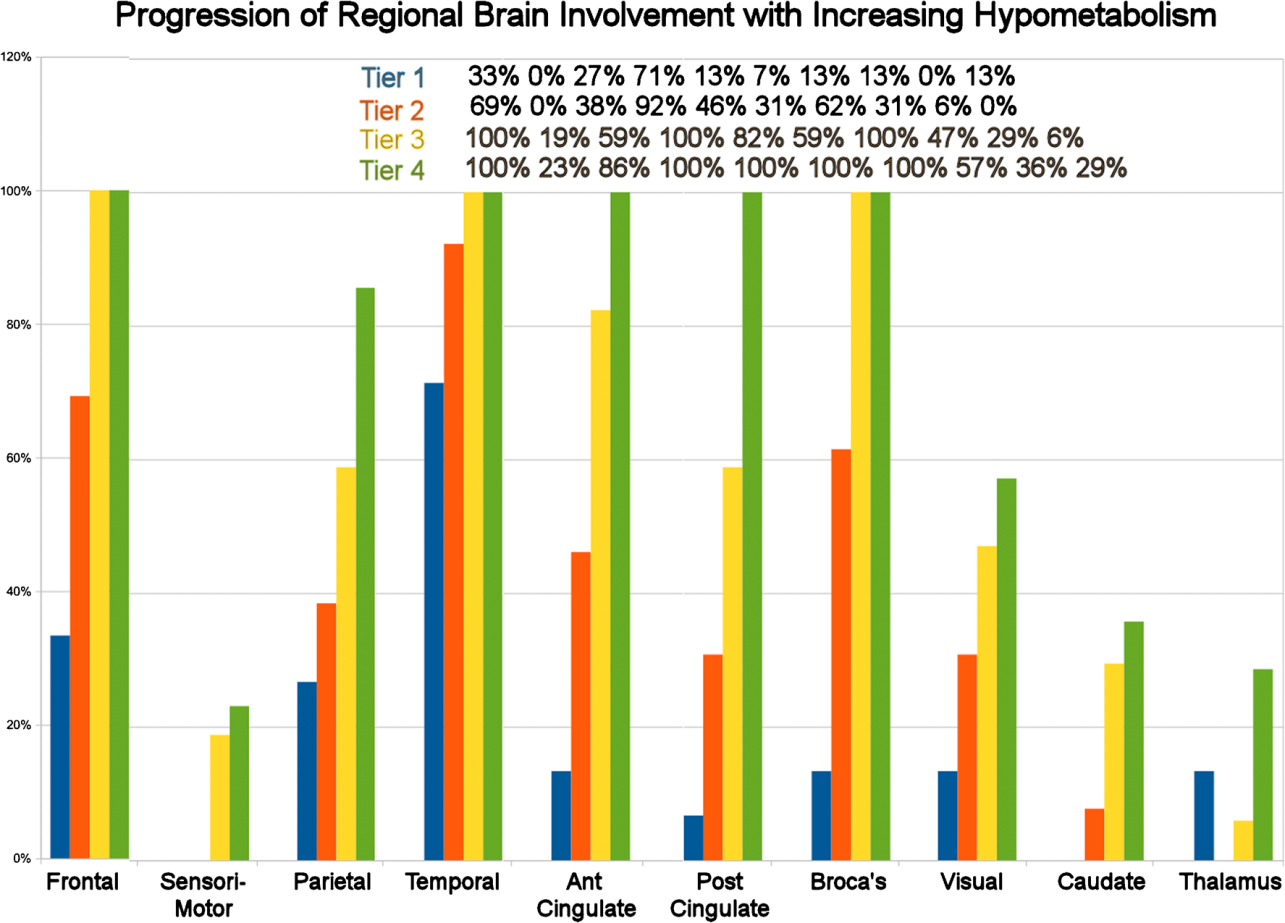
Progression of regional involvement by tier with increasing hypometabolism

Significant hypometabolism was present in all patients and across all types of joint replacement both MoM and MoP (Tables 2 and 4, Fig. 2). Major areas of hypometabolism retained their relative position for all patients (Fig. 3). All but seven patients had prominent hypometabolism of the right inferior lateral posterior temporal cortex.

**Table 4:** Range of hypometabolism in standard deviation for regions of the brain (min/median/max)

Tier	Range	Temporal	Frontal	Broca’s	Ant cingulate	Parietal	Post cingulate	Visual	Sensorimotor	Thalamus	Caudate
1	Min	− 1.66	− 1.69	− 1.97	− 1.75	− 1.66	− 1.72	− 2.08		− 1.70	
*N* = 14	Median	− 2.30	− 2.30	− 2.13	− 2.12	− 1.98		− 2.23		- 1.79	
	Max	− 3.55	− 2.62	− 2.28	− 2.49	− 3.10	− 1.72	− 2.38		− 1.87	
2	Min	− 1.66	− 1.68	− 1.65	− 1.67	− 1.66	− 1.76	− 1.77			− 1.65
*N* = 13	Median	− 2.14	− 2.05	− 1.98	− 2.01	− 2.10	− 1.85	− 2.31			− 1.65
	Max	− 3.68	− 3.01	− 2.96	− 3.81	− 3.43	− 2.43	− 2.55			
3	Min	− 1.67	− 1.65	− 1.71	− 1.73	− 1.67	− 1.71	− 1.70	− 1.73	− 1.78	− 1.71
*N* = 17	Median	− 2.16	− 2.15	− 2.30	-2.68	− 2.24	− 2.13	− 2.12	− 1.84		− 1.89
	Max	− 4.83	− 4.31	− 4.29	− 5.20	− 3.21	− 3.04	− 4.03	− 1.99	− 1.78	− 2.32
4	Min	− 1.68	− 1.66	− 1.77	− 1.69	− 1.69	− 1.77	− 1.75	− 1.66	− 1.69	− 2.03
*N* = 13	Median	− 2.67	− 2.48	− 3.15	− 3.06	− 2.33	− 2.77	− 2.18	− 1.67	− 1.79	− 2.06
	Max	− 6.49	− 4.81	− 4.59	− 5.65	− 3.90	− 3.92	− 6.29	− 2.21	− 2.15	− 2.45

Only three patients had known occupational exposure in the past to either heavy metal or solvents. In the tier one patients, one was a plumber, one a glazier, and another a dental hygienist who could have had exposure to mercury from dental amalgam preparation. In all, five additional patients had combat experience and five worked in heavy construction (Table 5).

**Table 5:** Potential toxic exposure with comparison MoCA and symptoms inventory scoring

Tier	Potential toxin exposure					MoCA		Symptoms inventory	
	Lead	Mercury	Solvent	Combat	Construction	No./Total	Range	No./Total	Range
1	1	1	1	1	1	7/14	23–29	14/14	3–12
2				1		7/13	22–28	13/13	3–10
3				3	2	5/17	22–28	17/17	11–22
4					1	4/13	23–28	13/13	17–28

There is no unique neurocognitive marker for heavy metal toxicity. In the initial full neurocognitive testing, findings were indeterminate often revealing only mild cognitive impairment. In one case, the PET scan of an excluded patient with the diagnosis of “dementia” was basically similar to all of the other scans in general. All patients with MoCA testing scored between 20 and 30. As such, the symptom inventory was considered a suitable adjunct for initial assessment (Table 5).

Of the 15 patients requiring revision surgery and removal of the cobalt component(s) from the affected joint due to ARMD, 8 had a positive response to removal of the cobalt alloy components. The post-revision scans were performed at about 6 months post-operative. The selection of the 6-month time frame for the post-revision FDG PET/CT brain scans was both anecdotal and from the limited published literature. Most patients felt better symptomatically at around 8 weeks post-op, while other reports were out to 3 and 6 months [15, 29–33] (Table 6). There is about a 10% latency of retained cellular cobalt at 1 year. [2, 34] Together, a reasonable initial time frame of 6 months was utilized but knowing that there had been no previous study to define a suitable timing for scanning.

**Table 6:** Published time frames for assay of cobalt levels post-revision surgery

Study	Pre-op	Post-op	7 weeks	8 weeks	3 months	6 months
Fritzsche	75 mcg/L			13 mcg/L		
Mao	410 nmol/L			60 nmol/L		
Mao	258 nmol/L			42 nmol/L		
Stepien	903.32 mcg/L					61.72 mcg/L
Woelber	108 mcg/L		0.7 mcg/L			
Harris		468.8 ppb		282.2 ppb	180 ppb	
Steens		398 mcg/L		25.4 mcg/L		< 1.0 mcg/L

**Fig. 4:**
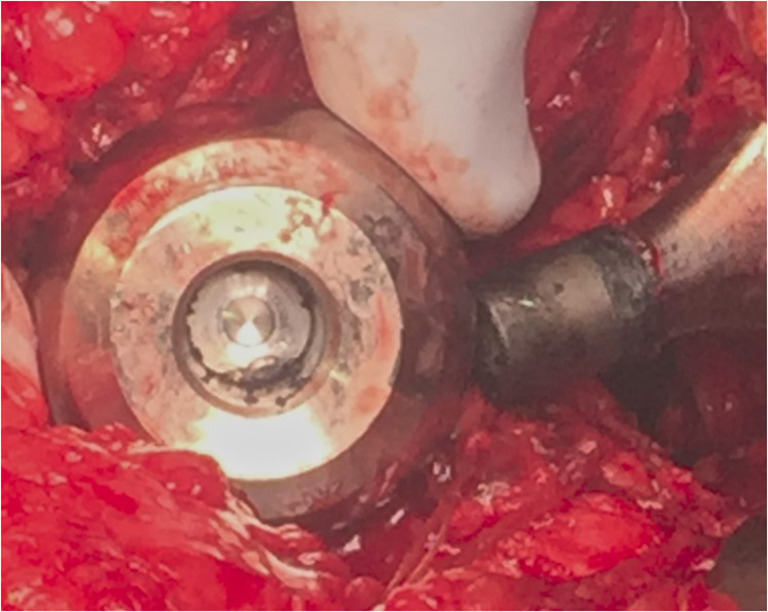
Marked corrosion (black) at the trunnion of the stem and the bore of the head

**Fig. 5:**
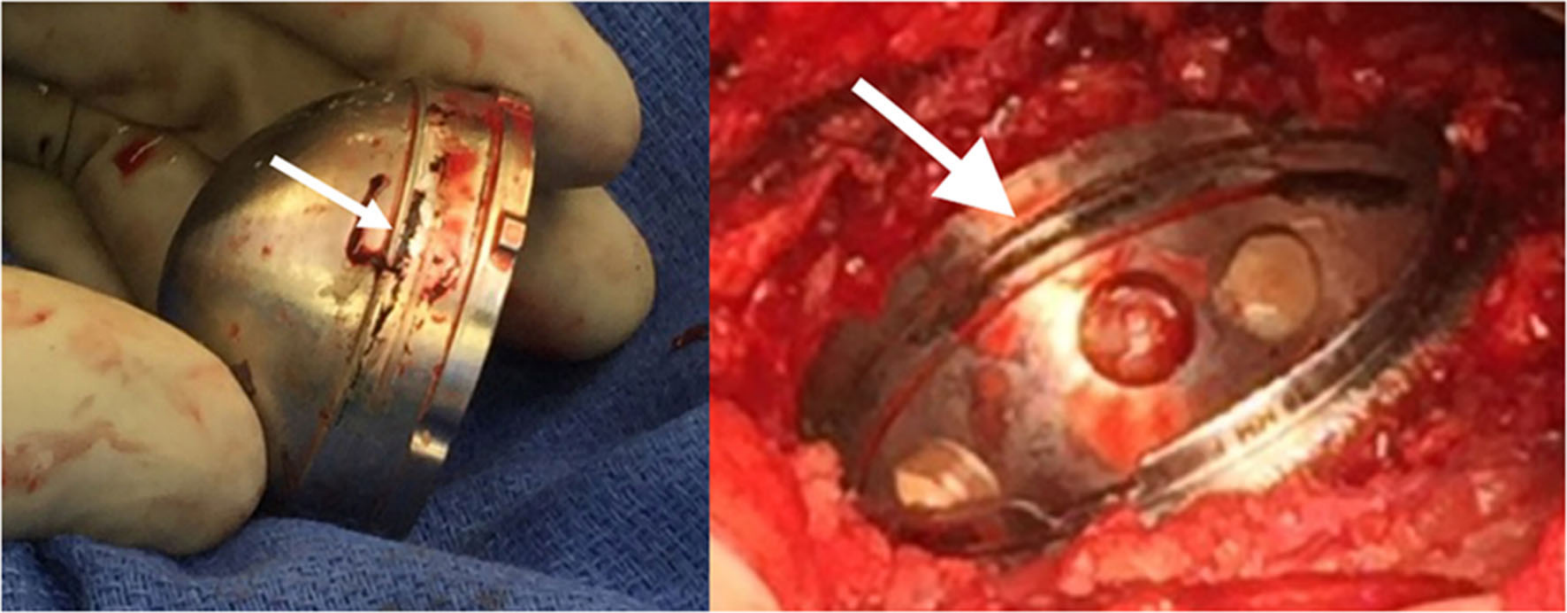
Intra-operative images showing band of corrosion (white arrows) of chrome cobalt acetabular liner (left) and socket (right)

Most patients had an approximate reduction of 50% in the number of regions and clusters of hypometabolism with significant improvement in two patients (Table 7). The intra-operative photographs demonstrated darkened corroded surfaces and how little metal may have been lost to create the systemic toxicity (Figs. 4 and 5).

**Table 7:** Changes in scoring, regions, and clusters with revision and removal of cobalt alloy component(s)

Patient	M/F	Age	Educational level	Type	Pre/post-revision	Summed hypometabolic score	Number of hypometabolic regions	Number of hypometabolic clusters
A	M	71	Federal Biologist	MoP	Pre	− 42.4	19	3
					Post	− 26.6	12	2
B	M	57	Retired Army Colonel	MoP	Pre	− 96.6	40	4
					Post	− 42	19	2
C	F	75	Unknown	MoP	Pre	− 226.9	80	16
					Post	− 131	53	10
D	M	52	B.A. Education	MoP	Pre	− 35.1	18	2
					Post	− 18.4	9	1
E	M	83	Railroad Engineer	MoP	Pre	− 228.4	90	22
					Post	− 88.74	34	6
F	F	67	Business owner	MoM	Pre	− 381.7	134	28
					Post	− 229.3	87	18
G	F	58	Military	MoP	Pre	− 23.6	11	1
					Post	− 15.4	8	1
H	M	58	Civil Engineer	MoM	Pre	− 277.3	111	23
					Post	− 38.8	19	4

## Vignettes

**Fig. 6:**
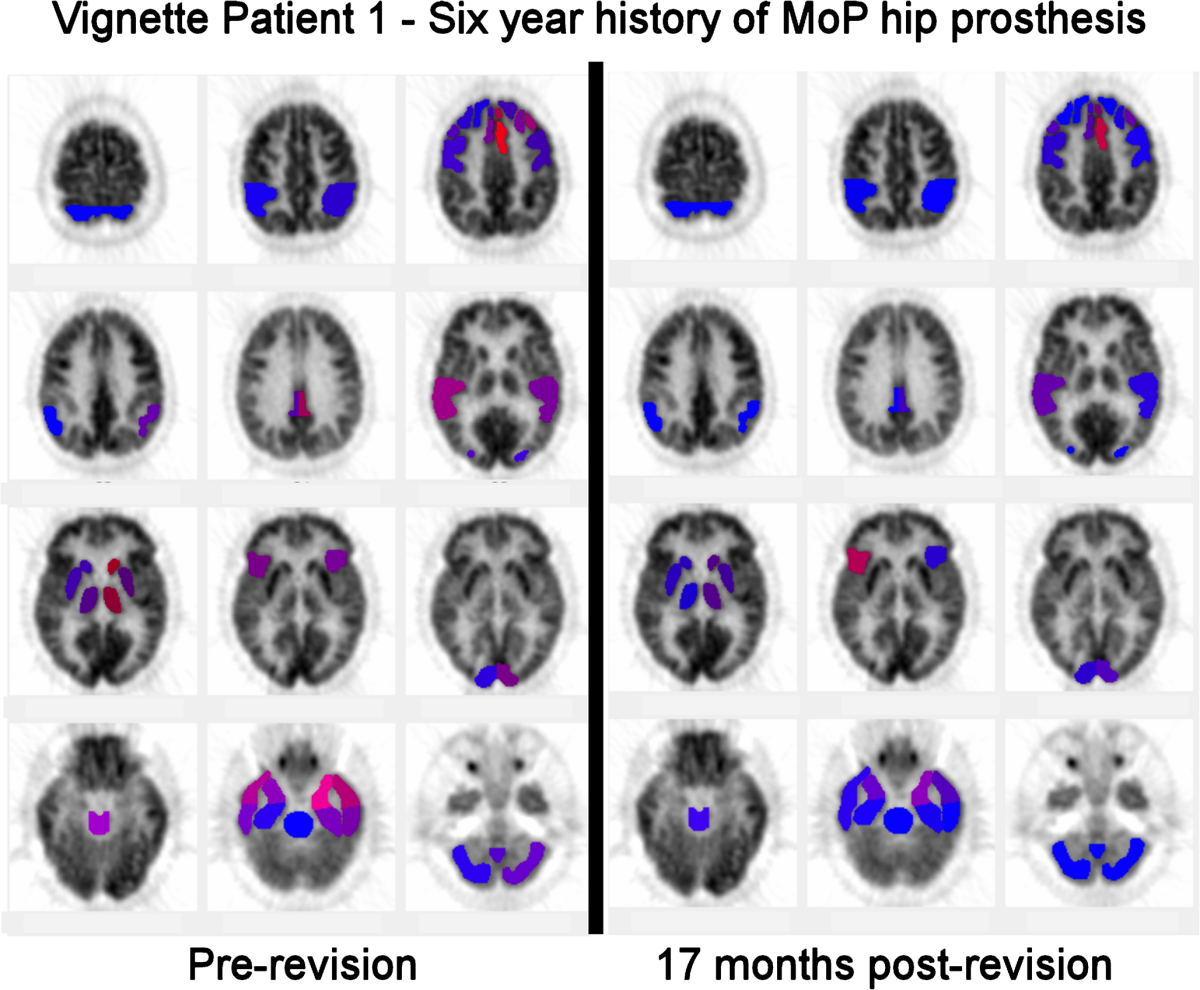
Vignette patient 1—pre- and post-revision NeuroQ scans showing interval improvement 17 months post-revision

Patient 1: An 83-year-old retired engineer with 6-year history of metal-on-plastic right hip prosthesis presented with new onset of bilateral restless leg syndrome, difficulty sleeping, sleep apnea, fatigue, exercise intolerance, dyspnea on exertion, decline in hearing, and changes in vision involving retinal dysfunction. Blood cobalt level was 4.8 mcg/L and urine cobalt was 49.1 mcg/L. Symptom score was 5/14. Metal-suppression MRI showed adverse reaction to metallic debris of the periprosthetic tissue including incomplete detachment of the gluteus medius tendon from the greater trochanter. Initial scan had a summed score of − 228.4 for 90 regions with 22 cluster areas of hypometabolism. CT showed mild symmetric prominence of sulci. Upon revision, there was marked corrosion at the trunnion of the stem and the bore of the head and the periprosthetic tissues were inflamed and thickened (Fig. 4). Cobalt level of right joint fluid collected at time of surgery was 1000 mcg/L and the chromium was 480 mcg/L. Repeat PET scan was 17 months post-revision. Repeat summed score for 34 regions was − 88.7 with 6 hypometabolic clusters (Fig. 6). At the time of the repeat scan, blood cobalt was 0.5 mcg/L and urine cobalt 2.5 mcg/L.

Patient 2: A 67-year-old retired business woman with 12-year history of MoM left hip prosthesis presented with ARMD. Blood cobalt level was 0.9 mcg/L and urine cobalt of 1.6 mcg/L. Symptom inventory score was 3/14. Metal-suppression MRI of hip detected possible pseudotumor. Initial scan unremarkable for age on CT with PET portion showing hypometabolism summed score of − 381.7 for 134 hypometabolic regions with 28 hypometabolic clusters. At surgery, corrosion found at taper interface between titanium alloy stem trunnion and cobalt head. Capsular tissue was consistent with ARMD. At the time of revision, patient was placed on oral N-acetyl-Cysteine (NAC) to further reduce cobalt burden. [35] At 13 months post-revision, repeat scan had 87 hypometabolic regions with score of − 229.6 and 18 hypometabolic clusters. Blood cobalt was 0.2 mcg/L and urine cobalt 0.4 mcg/L. Patient continues on N-acetyl-Cysteine.

Patient 3: A 57-year-old former army colonel with bilateral metal-on-plastic hip prostheses presented with pain. Right hip had no cobalt component. Previous revision left hip was 5 years prior for recalled Stryker Rejuvenate with replacement being CoP with cobalt-chromium-containing dual mobility liner. Blood cobalt was 1.5 mcg/L and urine cobalt 9.9 mcg/L. Symptom inventory was 7/14. CT imaging showed mild left temporal parietal sulcal prominence probably the result of prior IED trauma. While excluded from the primary list, his needed revision permitted assessment for response to therapy. There were 40 hypometabolic regions with score of − 96.6 and 4 hypometabolic clusters on original scan. Operative findings noted corrosion on the back side of the cobalt liner and acetabular shell interface (Fig. 5). Yellow-colored joint fluid at surgery had cobalt level of 220 mcg/L. PET scan at 8 months post-revision showed decrease to 19 hypometabolic regions, a score of − 42 and 2 hypometabolic clusters. At time of post-revision scan, urine cobalt was less than 1 mcg/L.

## Discussion

The regional susceptibility compares favorably with the distribution seen in Callender’s study on toxic exposure although differences are expected since organic compounds more readily cross the blood-brain barrier. In Callender’s study, the most commonly effected regions of hypometabolism in 33 encephalopathic workers from exposure to assorted neurotoxins were temporal (67.7%), frontal (61.3%), basal ganglia (45.2%), thalamus (29%), parietal (12.9), motor strip (9.68%), occipital (3.23%), and caudate (3.23%). [28]

No single laboratory or clinical finding had a direct proportional link to the brain scan findings. The scanned symptomatic patients in the study had significantly higher symptoms inventories than the cobalt normal group especially regarding tremor (Table 2). Individual tolerance to toxicity may be in part due to age, duration of exposure, baseline degree of cognitive function, and any coexistent neural disease process.

Apostoli reported severity of injury from high doses of cobalt which was related to dosage and duration of exposure in a study of auditory and optic toxicity in rabbits which confirmed his findings in humans**.** [36] Van Der Straeten has proposed a practical algorithm with four tiers of serum cobalt concentrations in the decision tree for monitoring with possible consideration for revision at levels greater than 20 mcg/L. [37] Such a measured approach may not take into account that toxicity may be present at lower levels, as noted here, or below presently accepted clinical thresholds.

The amount of metal loss needed to create systemic toxicity may be far less than presently expected with current blood cobalt level recommendations set at 7 to 10 mcg/L as threshold for concern. [38, 39] With the higher number of abnormal clusters, there may be convergence toward the pattern commonly seen with classic dementias and blurring discrimination from the latter. That said, cobalt toxicity will only worsen any clinical presentation. [15] The findings at lower summed scores and their accompanying abnormal clusters begin to demonstrate a pattern comparable with toxic encephalopathy rather than a classical dementia. [24] Also, 85% of the patients showed significant hypometabolism of the right inferior posterior lateral temporal lobe area.

The majority of patients undergoing revision with removal of cobalt alloy components had improvement (Table 7). This is a strong argument that cobalt was a significant contributor to the observed brain hypometabolism and a contributing source for the patients’ symptoms. For those that did not improve, the poor response may be due to a longer recovery time needed prior to post-revision scanning, the presence of residual cobalt debris in the joint, confounding concurrent disease, and/or irreversible progression of cobalt-accentuated encephalopathy or dementia.

## Conclusion

All of the scanned patients had regions of significant hypometabolism. In patients with elevated systemic cobalt levels and clinical presentation of toxicity, regional areas of involvement (greatest to least) were as follows: temporal lobes, frontal lobes, anterior cingulate gyri, parietal lobes, posterior cingulate gyri, visual cortex, thalamic areas, and lastly caudate. Of patients, 85% had significant hypometabolism of the right posterior inferior lateral temporal cortex.

Cobalt toxicity may be more common and present at lower concentrations than appreciated in the current literature. Any joint prostheses with a cobalt alloy component may place the patient at risk for cobalt toxicity. F-18 FDG PET brain imaging may provide an early and reproducible adjunct to the clinical assessment of patients with elevated cobalt levels and systemic symptoms of cobalt toxicity. Individuals with failing prostheses may incur delayed treatment or no treatment due to misdiagnosis or underestimation of the extent of neurotoxicity. Laboratory tests may provide a simple and inexpensive method for monitoring systemic cobalt levels, but may not adequately measure the metabolic burden from cobalt neurotoxicity. Quantitative F-18 FDG PET brain imaging may aid in the decision process as to treatment options and timing for potential medical versus surgical intervention.

Further studies with PET brain imaging are needed to establish a safer threshold for exposure, help define monitoring protocols for patients with cobalt alloy prostheses, identify reversible thresholds for toxicity, and better discriminate cobalt from confounding unrelated illness. Considering the overlap of older individuals with prosthetic joint replacement and cognitive decline, additional attention as to potential contribution of cobalt toxicity may be beneficial in any study. This use of quantitative metabolic imaging may become a template for redefining thresholds for other known neurotoxins.

With imaging findings similar to those reported for chronic toxic encephalopathy (CTE), a descriptive acronym for cobalt alloy related brain toxicity may be arthroprosthetic cobalt encephalopathy (ACE).
